# Slug expression is an independent prognostic parameter for poor survival in colorectal carcinoma patients

**DOI:** 10.1038/sj.bjc.6603193

**Published:** 2006-06-13

**Authors:** M Shioiri, T Shida, K Koda, K Oda, K Seike, M Nishimura, S Takano, M Miyazaki

**Affiliations:** 1 Department of General Surgery, Chiba University Graduate School of Medicine, Chiba 260-8670, Japan; 2 Department of Academic Surgery, Chiba University Graduate School of Medicine, Chiba 260-8670, Japan

**Keywords:** Slug, E-cadherin, colorectal carcinoma, prognosis

## Abstract

Slug, a member of the Snail family of transcription factors, plays a crucial role in the regulation of epithelial-mesenchymal transition (EMT) by suppressing several epithelial markers and adhesion molecules including E-cadherin. Recently, several studies have reported Slug to be expressed in breast carcinoma, oesophageal carcinoma accompanied with shorter survival. In this study, we first investigated expression of Slug mRNA in five colorectal carcinoma cell lines by reverse transcription–polymerase chain reaction. Furthermore, we investigated Slug and E-cadherin expression by immunohistochemistry in 138 patients with colorectal carcinoma. Slug mRNA was clearly expressed in four out of five colorectal carcinoma cell lines. Positive expression of Slug and E-cadherin was observed in 37 and 58% of cases, respectively. The positive expression of Slug was significantly associated with Dukes stage and distant metastasis (*P*=0.0027 and 0.0007), and the positive expression of Slug had a significant impact on patient overall survival (*P*<0.0001, log-rank test). Moreover, patients with positive expression of Slug and reduced expression of E-cadherin showed the worst prognosis (*P*<0.0001, log-rank test). Multivariate analysis indicated that Slug expression was an independent prognostic factor. These results suggest that positive Slug expression in colorectal carcinoma patients may become a significant parameter of poor prognosis.

Colorectal carcinoma is among the most frequent malignant diseases worldwide, and is one of the leading causes of cancer-related deaths (Friedlich and Stem, 2000). A significant number of patients with colorectal carcinoma who undergo apparently curative operation unfortunately develop local recurrence or distant metastasis leading to shorter survival (Rosen *et al*, 1998). Therefore, adjuvant therapy is needed after surgical resection for such biologically aggressive colorectal carcinomas. For this reason identification of factors that accurately predict prognosis in colorectal carcinoma is strongly required. A deeper insight into the carcinogenesis and the factors related to the aggressiveness of colorectal carcinoma may be necessary for this requirement. Several previous works have revealed that epithelial-mesenchymal transition (EMT) plays a crucial role in the progression and aggressiveness of colorectal carcinoma (Barker and Clevers, 2001; Bates and Mercurio, 2005; Brabletz *et al*, 2005).

Slug (SNAI2), a member of the Snail family of zinc-finger transcription factors was first identified in the neural crest and developing mesoderm in the chick embryos (Nieto *et al*, 1994). It is highly expressed in cells undergoing EMT in the developing chick embryo, where it is critical in the formation of the primitive streak, endocardial cushions, decondensing somites and in closure of the palate. Recently, Snail family transcription factors have been reported to repress E-cadherin expression, which mediates cell-to-cell adhesion, and increase cancer invasion in various malignancies (Battle *et al*, 2000; Cano *et al*, 2000). The Snail transcription factors include Snail (SNAI1) and Slug both of which have been implicited in various malignancies. For example, previous work has revealed that Slug to be the *in vivo* repressor of E-cadherin associated with poor prognosis in breast carcinoma and oesophageal squamous cell carcinoma (Hajra *et al*, 2002; Martin *et al*, 2005; Uchikado *et al*, 2005).

In colorectal carcinoma, it has been reported that there is a frequent loss of E-cadherin, which the majority of them (approximately 80%) are due to promoter hypermethylation (Garinis *et al*, 2002). However, the remaining mechanisms that control the loss of E-cadherin in colorectal carcinoma is not yet well understood. Therefore, Snail transcription factors may be involved in the regulation of E-cadherin in some of the colorectal carcinomas. Moreover, there are only few reports concerning the role of Snail family transcription factors in colorectal carcinoma. Previous study revealed that Snail was positive in 78% of colorectal carcinoma patients, however, the functional consequences of Snail overexpression was not determined either biologically or clinically in that study (Roy *et al*, 2005). Furthermore, to our knowledge, there are no reports concerning the expression of Slug in colorectal carcinoma.

The purpose of the present study was to examine the *in vivo* significance of Slug in colorectal carcinoma and the correlation between Slug and E-cadherin expression in colorectal carcinoma. Also, to clarify whether Slug may be used as a novel parameter to predict prognosis in colorectal carcinoma.

## MATERIALS AND METHODS

### Cell lines

The human colon adenocarcinoma cell lines (DLD1, HT29, WiDr, Colo320DM, SW620) obtained from American Type Culture Collection (Rockville, MD, USA). All the cell lines were maintained in RPMI 1640 medium supplemented with 10% FCS, 1% penicillin and streptomycin at 37°C in 5% CO_2_.

### Reverse transcription-polymerase chain reaction

Total RNA from the cell lines were obtained using RNeasy Mini kit (Qiagen, Tokyo, Japan) according to the manufacture's instructions. Expression of Slug was examined by reverse transcription–polymerase chain reaction (RT–PCR) with the use of the following primers: forward 5′-GTG ATT ATT TCC CCG TAT CTC TAT-3′, reverse 5′-CAA TGG CAT GGG GGT CTG AAA G-3′ (Yokoyama *et al*, 2001). For control, *β*-actin cDNA was amplified. The condition of PCR were: initial denaturing at 95°C for 10 min, followed by 38 cycles of denaturing at 94°C for 60 s, annealing at 53°C for 60 s and extension at 72°C for 90 s. All PCR products were visualized by electrophoresis and ethidium bromide staining in 2% agarose gels. RT–PCR was performed in a triplicate.

### Patients and tissues

Formalin-fixed and paraffin-embedded samples from primary colorectal carcinoma (*n*=138) were obtained from Department of General Surgery, Chiba University Hospital, Japan, from 1996 to 2000. The patient age ranged from 26 to 85 years old. Patients who underwent neoadjuvant therapy, treated by endoscopic mucosal resection, and with carcinomas besides colorectal were excluded. Written informed consent was obtained from each patient. Clinical and pathological data were documented and entered into a specific tumour registry after surgery and follow-up (Table 1). The median follow-up period was 5.11 years. This study was designed as historical prospective study.

### Immunohistochemistry

The specimens were cut into 3-*μ*m-thick sections, which were mounted on glass slides. Immunohistochemical staining was performed using labeled streptoavidine-biotin-peroxidase and microwave antigen retrieval technique. Monoclonal antibody against E-cadherin (Takara Biotechnology Inc., Takara, CA, USA) and polyclonal antibody against Slug (D-19, Santa Cruz Biotechnology Inc., Santa Cruz, CA, USA) were used in the study. Working dilutions of E-cadherin and Slug were both 1 : 100. The specificity and sensitivity of Slug antibody was confirmed by Western blotting and RT–PCR (Data not shown). Negative controls were performed by omission of the primary antibody. The tissue sections were washed in water, counterstained with Mayer's haematoxylin. Immunohistochemistry was performed in a duplicate.

### Evaluation of immunohistochemistry

Expression of E-cadherin was determined as previous study (Shiozaki *et al*, 1991). The tumour cells that stained as strongly as normal epithelial cells were considered as preserved expression, and those which exhibited weaker staining than normal epithelial cells or completely showed negative staining were considered as reduced expression. Expression of Slug was determined as positive when cytoplasmic and/or perinuclear staining was seen in more than 10% of the tumour cells (Uchikado *et al*, 2005). Expression of Slug was considered negative when no or less than 10% of the tumour cells were stained. In the evaluation of these two molecules high-power field (×200) of 10 random areas (within the tumour) were selected. Evaluation of immunohistochemistry was independently performed by two investigators (MS and TS).

### Statistical analysis

Overall survival rates were calculated according to the Kaplan–Meier method. Differences between the groups were evaluated using the *χ*^2^ test, Student's *t* test, and the log-rank test. The prognostic factors were examined by univariate and multivariate analyses (Cox's proportional hazards model). Results were considered significant when *P*<0.05 was obtained. All the statistical analyses were performed using SPSS 11.5 for Windows (SPSS Inc., Chicago, IL, USA).

## RESULTS

### Expression of Slug mRNA in colorectal carcinoma cell lines

**Figure 1:**
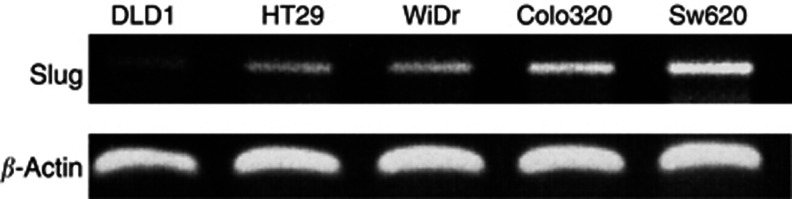
Expression of Slug mRNA in colorectal carcinoma cell lines; DLD1, HT29, WiDr, Colo320DM, SW620. Reverse transcription-polymerase chain reaction was performed and PCR product samples were subjected to 2% agarose gel electrophoresis and visualized by staining with ethidium bromide.

RT–PCR showed Slug mRNA expression in HT-29, WiDr, Colo320DM, and SW620 but showed faint expression in DLD1. SW620 showed the strongest expression among all the cell lines (Figure 1).

### Expression of Slug and E-cadherin in colorectal carcinoma by immunohistochemistry

**Figure 2:**
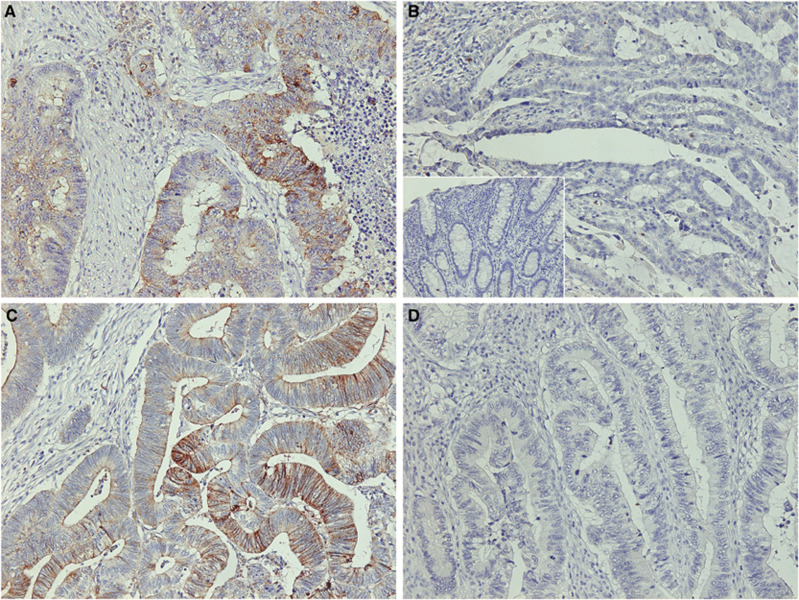
An immunohistochemical analysis for Slug and E-cadherin in colorectal carcinoma. (**A**) Positive expression of Slug in the tumour cells (×200). (**B**) Negative expression of Slug in the tumour cells (×200). Inset, negative staining in the normal mucosa (×200). (**C**) Preserved E-cadherin expression is detected at the cell–cell borders and the cell membrane (×200). (**D**) Example of reduced expression of E-cadherin (×200).

Slug was observed mainly in the cytoplasm of the tumour cells. Expression of Slug was considered as positive in 37% of all patients (51 of 138; Figure 2A and B). Slug was not expressed in the normal mucosa (Figure 2B, Inset). Expression of E-cadherin was observed on the cell membrane and borders of the cancer cells. E-cadherin was considered as preserved in 58% of all patients (80 of 138; Figure 2C and D).

### Relationship between Slug and E-cadherin expression and clinico-pathologic data

Slug expression was significantly associated with Dukes stage and distant metastasis (*P*=0.0027, 0.0007, respectively; Table 1). E-cadherin expression was significantly associated with depth of tumour, lymph node metastasis, and Dukes stage (*P*=0.0103, 0.0069, and 0.0054, respectively; Table 1).

In the present study, Slug expression was not correlated with E-cadherin expression (*P*=0.3594, Table 2).

### Prognostic value of Slug and E-cadherin expression in all patients (Dukes A∼D)

**Figure 3:**
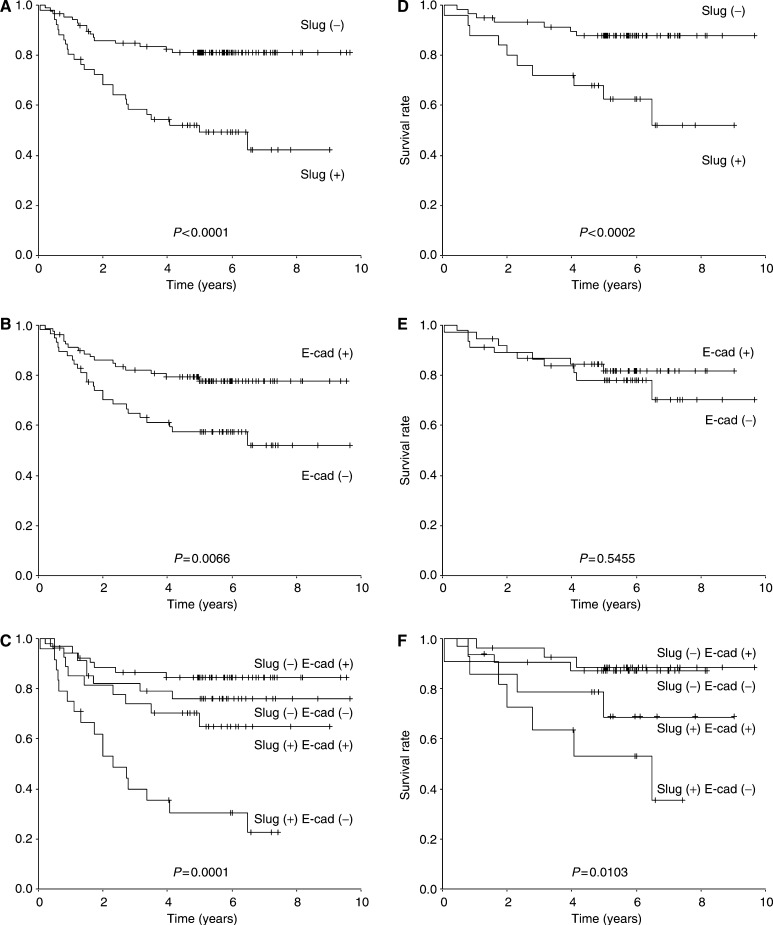
(**A**) Overall survival of all patients (Dukes A–D) in relation to the expression of Slug (*P*<0.0001). (**B**) Overall survival of all patient's (Dukes A–D) in relation to the expression of E-cadherin (*P*=0.0066). (**C**) Overall survival of all patient's (Dukes A–D) in relation to the combination of Slug and E-cadherin expression (*P*<0.0001). (**D**) Overall survival of Dukes B, C patient's in relation to the expression of Slug (*P*=0.0002). (**E**) Overall survival of Dukes B, C patient's in relation to the expression of E-cadherin (*P*=0.5455). (**F**) Overall survival of Dukes B, C patient's in relation to the combination of Slug and E-cadherin expression (*P*=0.0103). Slug(+); positive expression of Slug, Slug(−); negative expression of Slug, E-cad(+); preserved E-cadherin expression, E-cad(−); reduced E-cadherin expression.

Patients with Slug positive expression (*n*=51) survived significantly shorter than those with negative expression (*n*=87) (*P*<0.0001; Figure 3A). Patients with reduced E-cadherin (*n*=58) survived significantly shorter than those with preserved E-cadherin expression (*n*=80) (*P*=0.0066; Figure 3B). Patients with combined Slug positive expression and reduced E-cadherin expression (*n*=24) survived significantly shorter than those with other combinations (*P*<0.0001; Figure 3C).

### Prognositic value of Slug and E-cadherin expression in Dukes B, C patients

With regard to Dukes B, C patients (*n*=84), patients with positive Slug expression (*n*=25) showed a significant shorter survival than those with negative expression (*n*=59) (*P*=0.039; Figure 3D). However, there was no statistical significance between patients with reduced E-cadherin (*n*=38) and preserved E-cadherin patients (*n*=46) (*P*=0.5455; Figure 3E). Patients with combined Slug positive expression and reduced E-cadherin expression (*n*=11) survived significantly shorter than those with other combinations (*P*=0.0103; Figure 3F).

### Univariate and multivariate analyses of survival

The univariate and multivariate analyses of factors related to patient prognosis were performed in all patients (*n*=138, Dukes A∼D). The univariate analysis showed: age, E-cadherin expression, Slug expression, lymph node metastasis, venous invasion, lymphatic invasion, distant metastasis, and Dukes stage were significantly associated with patient survival (*P*<0.05, Table 3). All the variables, which showed *P*<0.05 by the univariate analyses were used for multivariate analyses. Multivariate analysis using the Cox's proportional hazards model revealed that Slug expression, E-cadherin expression, lymph node metastasis, and distant metastasis were independent and significant prognostic factors in all patients (*n*=138, Dukes A∼D) (Table 4).

Moreover, the multivariate analysis of survival with regard to Dukes B, C patients (*n*=84) revealed that expression of Slug was the only independent prognostic factor when adjusted for age, expression of E-cadherin, lymph node metastasis, lymphatic invasion, and vessel invasion (Table 5).

## DISCUSSION

Carcinoma is a complex disease of differentiation, tissue organization, and altered growth. These cellular activities are important for normal embryonic development and maintenance of proper function and structure of the mature organism. When these factors disrupt, as evidenced in carcinoma, it results in loss of tissue differentiation and facilitates invasion and metastasis. The EMT plays an important role in the acquisition of invasive and aggressive phenotype of carcinoma. Therefore, loss of expression of epithelial markers and adhesion molecules including E-cadherin is important in cancer progression and development. Indeed several studies have clarified the role of EMT in metastasis of colorectal carcinoma (Barker and Clevers, 2001).

Recently family of the Snail transcription factors (Slug and/or Snail) has been found to repress E-cadherin and contribute to tumour progression in various malignancies such as hepatocellular carcinoma (Sugimachi *et al*, 2003; Miyoshi *et al*, 2004), breast carcinoma (Hajra *et al*, 2002), melanoma (Poser *et al*, 2001), oral squamous cell carcinoma (Yokoyama *et al*, 2001), and oesophageal squamous cell carcinoma (Uchikado *et al*, 2005).

In colorectal carcinoma, previous study revealed that reduction of E-cadherin is seen approximately in 38–46% of the patients accompanied by significant poor survival, which is consistent in part with our present study (Mohri, 1997; Ghadimi *et al*, 1999; Karatzas *et al*, 1999; Ikeguchi *et al*, 2000; Aoki *et al*, 2003). Furthermore, it has been reported that majority of the E-cadherin reduction (approximately 80%) is due to promoter hypermethylation but not as a result of mutations or LOH at the E-cadherin gene locus in colorectal carcinoma (Hyas *et al*, 1997, Garinis *et al*, 2002). Present study showed reduction of E-cadherin was significantly correlated with tumour stage, lymph node metastasis, and advanced Dukes stage (Table 1). Also, multivariate analysis among all the patients (Dukes A∼D, *n*=138) revealed reduction of E-cadherin as one of the independent prognostic factors (Table 4). However, E-cadherin reduction showed no statistical significance in the overall survival with regard to Dukes B, C patients (*n*=84) in our study (Figure 3E).

In the present study, we first examined the expression of Slug mRNA *in vitro* using five human colorectal carcinoma cell lines. The result that four out of five cell lines (HT29, WiDr, Colo320DM, SW620) showed positive expression of Slug mRNA prompted us to investigate the *in vivo* expression of Slug in colorectal carcinoma. Immunohistochemical staining revealed that Slug was positively expressed in 37% of the patients, which was significantly associated with distant metastasis and Dukes stage. Furthermore, the overall survival of patients with Slug positive expression was significantly poorer than those with negative expression. These *in vivo* results seems to support our *in vitro* study that SW620 showed the strongest expression of Slug mRNA, because SW620 is an aggressive metastatic colorectal carcinoma cell line (Trainer *et al*, 1988).

Although expression of Snail was not investigated in the present study, previous study revealed that positive expression of Snail was seen in a majority of colorectal carcinoma patients (78%), which showed no significant correlation with any of the clinicopathological factors except the patient's age (Roy *et al*, 2005). These findings may suggest that transcription factor Slug rather than Snail plays a crucial role in tumour invasion, metastasis, and progression of colorectal carcinoma and may contribute to aggressive phenotype. Moreover, the result that majority of colorectal carcinoma express Snail and those with aggressive behaviour express Slug, implies that Snail upregulation may be involved in the early progression phase, and Slug upregulation may be acquired in the latter progression phase of colorectal carcinogenesis. The biological difference and role of Slug and Snail in colorectal carcinoma needs to be clarified in the future.

To our knowledge, this is the first report concerning the clinical significance of Slug expression in colorectal cancer. In the present study, although Slug was detected in some tumours where E-cadherin expression was decreased, positive Slug expression was not correlated with reduced E-cadherin expression. This may be because in colorectal casrcinoma the majority of E-cadherin reduction is due to promoter hypermethylation (Garinis *et al*, 2002). On the other hand, previous study using a melanoma cell line showed mutated E-cadherin cause downregulation of Slug *in vitro* (Laux *et al*, 2004). The relationship between Slug expression and tumour associated E-cadherin reduction in colorectal carcinoma needs further investigation.

Our results suggest that Slug itself may participate in progression and aggressiveness of colorectal carcinoma, not just owing to the repression of E-cadherin. In the previous study, Slug has been reported to have an antiapoptotic effect on leukaemia cells and breast carcinoma cells (Inukai *et al*, 1999; Hemavathy *et al*, 2000; Kajita *et al*, 2004) and is capable to down regulate several epithelial markers involved in cell–cell adhesion such as cytokeratin18, muc-1, desmoplakin, occludin, and claudin-1 (Cano *et al*, 2000; Guaita *et al*, 2002; Kajita *et al*, 2004; Martinez-Estrada *et al*, 2005). Moreover, previous study has shown that Slug-overexpressing mice developed mesenchymal tumours, mainly leukaemias and sarcomas (Perez-Mancera *et al*, 2005). These findings indicate that Slug plays a certain role in the carcinogenesis of mesenchymal tumours and is capable to contribute to the invasiveness of human malignancies not just by repressing E-cadherin but implicating in EMT accompanied by downregulation of several epithelial markers and enhanced cell survival.

In the present study, tumours with both increased expression of Slug and reduced expression of E-cadherin showed the worst prognosis, and the tumours with the opposite expression showed the best prognosis. Although the patient samples included in the present study were limited, univariate and multivariate analyses revealed Slug to be an independent prognostic factor in colorectal carcinoma patients. Furthermore, with regard to Dukes B and C patients, multivariate analysis revealed Slug to be the only independent prognostic factor. These results strongly indicate that Slug expression might be a novel parameter to predict prognosis for the aggressiveness of colorectal carcinoma and the combination of Slug and E-cadherin expression might give us precise prognostic information in colorectal carcinoma including those which curative operation have been performed. Expression of Slug may also provide useful information in selecting patients who need adjuvant therapy and strict surveillance. Slug may be an attractive target for the treatment of colorectal carcinoma.

## Figures and Tables

**Table 1:** Slug and E-cadherin expression in relation to clinicopathologic findings

		**E-cadherin**			**Slug**		
		**Preserved**	**Reduced**		**Positive**	**Negative**	
**Characteristics**	**Total**	**80 (58.0%)**	**58 (42.0%)**	***P***	**51 (37.0%)**	**87 (63.0%)**	***P***
*Age*				0.5068			0.8861
	63.16±12.09	62.58±11.51	63.97±12.90		63.35±13.31	63.05±11.39	
							
*Gender*				0.0854			0.8082
Male	83 (60.1)	53 (66.3)	30 (51.7)		30 (58.8)	53 (60.9)	
Female	55 (39.9)	27 (33.7)	28 (48.3)		21 (41.2)	34 (39.1)	
							
*Tumour location*				0.1123			0.7425
Colon	89 (64.5)	56 (70.0)	33 (56.9)		32 (62.7)	57 (65.5)	
Rectum	49 (35.5)	24 (30.0)	25 (43.1)		19 (37.3)	30 (34.5)	
							
*Histology*				0.2878			0.7484
Well	28 (20.3)	16 (20.0)	12 (20.7)		8 (15.7)	20 (23.0)	
Mod	75 (54.3)	48 (60.0)	27 (46.6)		30 (58.8)	45 (51.7)	
Por	9 (6.5)	5 (6.3)	4 (6.9)		3 (5.9)	6 (6.9)	
Others	26 (18.9)	11 (13.7)	15 (25.8)		10 (19.6)	16 (18.4)	
							
*pT*				0.0103			0.1443
T1	10 (7.2)	10 (12.5)	0 (0.0)		2 (3.9)	8 (9.2)	
T2	19 (13.8)	14 (17.5)	5 (8.6)		6 (11.8)	13 (14.9)	
T3	86 (62.3)	45 (56.3)	41 (70.7)		30 (58.8)	56 (64.4)	
T4	23 (16.7)	11 (13.7)	12 (20.7)		13 (25.5)	10 (11.5)	
							
*pN*				0.0069			0.4604
N0	77 (55.8)	51 (63.8)	26 (44.8)		29 (56.9)	48 (55.2)	
N1	47 (34.1)	26 (32.5)	21 (36.2)		15 (29.4)	32 (36.8)	
N2	14 (10.1)	3 (3.7)	11 (19.0)		7 (13.7)	7 (8.0)	
							
*pM*				0.0664			0.0007
M0	108 (78.3)	67 (83.8)	41 (70.7)		32 (62.7)	76 (87.4)	
M1	30 (21.7)	13 (16.2)	17 (29.3)		19 (37.3)	11 (12.6)	
							
*Dukes*				0.0054			0.0027
A	24 (17.4)	21 (26.3)	3 (5.2)		7 (13.7)	17 (19.5)	
B	45 (32.6)	27 (33.7)	18 (31.0)		17 (33.3)	28 (32.2)	
C	39 (28.3)	19 (23.8)	20 (34.5)		8 (15.7)	31 (35.7)	
D	30 (21.7)	13 (16.2)	17 (29.3)		19 (37.3)	11 (12.6)	
							
*Lymphatic invasion*				0.6164			0.1486
Negative	75 (54.3)	45 (56.3)	30 (51.7)		23 (45.1)	52 (59.8)	
Positive	61 (44.2)	34 (42.5)	27 (46.6)		26 (51.0)	35 (40.2)	
Unknown	2 (1.5)	1 (1.2)	1 (1.7)		2 (3.9)	0 (0.0)	
							
*Venous invasion*				0.8944			0.1189
Negative	97 (70.2)	56 (70.0)	41 (70.7)		31 (60.8)	66 (75.9)	
Positive	39 (28.3)	23 (28.8)	16 (27.6)		18 (35.3)	21 (24.1)	
Unknown	2 (1.5)	1 (1.2)	1 (1.7)		2 (3.9)	0 (0.0)	

**Table 2:** Comparison of Slug expression and E-cadherin expression

	**E-cadherin expression**		
**Slug expression**	**Preserved (*n*=80)**	**Reduced (*n*=58)**	***P***
Positive (*n*=51)	27	24	0.3594
Negative (*n*=87)	53	34	

**Table 3:** Univariate Cox's proportional hazard analysis in all patients (Dukes A–D, *n*=138)

	**Overall survival**		
**Prognostic factors**	**HR**	**95% CI**	P
E-cadherin	2.294	(1.238, 4.252)	0.0083
Slug	3.367	(1.803, 6.288)	0.0001
Age	1.029	(1.001, 1.057)	0.0395
Gender	1.169	(0.622, 2.199)	0.6270
Tumour differentiation (well *vs* others)	0.475	(0.187, 1.209)	0.1183
tumour location (colon *vs* rectum)	1.432	(0.744, 2.756)	0.2878
Distant metastasis	10.259	(5.438, 19.356)	<0.0001
Lymph node metastasis	4.283	(2.150, 8.534)	<0.0001
Lymphatic invasion	4.531	(2.264, 9.070)	<0.0001
Vessel invasion	2.848	(1.895, 4.280)	<0.0001

HR=hazard ratio, CI=confidence interval.

**Table 4:** Multivariate Cox's proportional hazard analysis in all patients (Dukes A–D, *n*=138)

	**Overall survival**		
**Prognostic factors**	**HR**	**95% CI**	***P***
Age	1.024	(0.998, 1.052)	0.0708
E-cadherin	2.249	(1.164, 4.343)	0.0158
Slug	2.212	(1.127, 4.342)	0.0210
Distant metastasis	5.232	(2.550, 10.733)	<0.0001
Lymph node metastasis	2.691	(1.280, 5.658)	0.0090
Lymphatic invasion	1.922	(0.824, 4.484)	0.1308
Vessel invasion	1.943	(0.935, 4.041)	0.0752

HR=hazard ratio, CI=confidence interval.

**Table 5:** Multivariate Cox's proportional hazard analysis in Dukes B, C patients (*n*=84)

	**Overall survival**		
**Prognostic factors**	**HR**	**95% CI**	***P***
Age	1.035	(0.990, 1.082)	0.1259
E-cadherin	1.668	(0.616, 4.516)	0.3141
Slug	3.260	(1.135, 9.368)	0.0282
Lymph node metastasis	2.097	(0.736, 5.972)	0.1655
Lymphatic invasion	1.831	(0.580, 5.781)	0.3025
Vessel invasion	2.503	(0.783, 8.005)	0.1219

HR=hazard ratio, CI=confidence interval.
